# ASM-024, a Piperazinium Compound, Promotes the *In Vitro* Relaxation of β2-Adrenoreceptor Desensitized Tracheas

**DOI:** 10.1371/journal.pone.0120095

**Published:** 2015-03-23

**Authors:** Evelyne Israël-Assayag, Marie-Josée Beaulieu, Yvon Cormier

**Affiliations:** 1 Institut universitaire de cardiologie et de pneumologie de Québec (IUCPQ), Québec, Québec, Canada; 2 Asmacure Ltée, Québec, Québec, Canada; Cinvestav-IPN, MEXICO

## Abstract

Inhaled β2-adrenoreceptor agonists are widely used in asthma and chronic obstructive pulmonary disease (COPD) for bronchoconstriction relief. β2-adrenoreceptor agonists relax airway smooth muscle cells via cyclic adenosine monophosphate (cAMP) mediated pathways. However, prolonged stimulation induces functional desensitization of the β2-adrenoreceptors (β2-AR), potentially leading to reduced clinical efficacy with chronic or prolonged administration. ASM-024, a small synthetic molecule in clinical stage development, has shown activity at the level of nicotinic receptors and possibly at the muscarinic level and presents anti-inflammatory and bronchodilator properties. Aerosolized ASM-024 reduces airway resistance in mice and promotes *in-vitro* relaxation of tracheal and bronchial preparations from animal and human tissues. ASM-024 increased *in vitro* relaxation response to maximally effective concentration of short—acting beta-2 agonists in dog and human bronchi. Although the precise mechanisms by which ASM-024 promotes airway smooth muscle (ASM) relaxation remain unclear, we hypothesized that ASM-024 will attenuate and/or abrogate agonist-induced contraction and remain effective despite β2-AR tachyphylaxis. β2-AR tachyphylaxis was induced with salbutamol, salmeterol and formoterol on guinea pig tracheas. The addition of ASM-024 relaxed concentration-dependently intact or β2-AR desensitized tracheal rings precontracted with methacholine. ASM-024 did not induce any elevation of intracellular cAMP in isolated smooth muscle cells; moreover, blockade of the cAMP pathway with an adenylate cyclase inhibitor had no significant effect on ASM-024-induced guinea pig trachea relaxation. Collectively, these findings show that ASM-024 elicits relaxation of β2-AR desensitized tracheal preparations and suggest that ASM-024 mediates smooth muscle relaxation through a different target and signaling pathway than β2-adrenergic receptor agonists. These findings suggest ASM-024 could potentially provide clinical benefit when used adjunctively with inhaled β2-adrenoreceptor agonists in those patients exhibiting a reduced response to their chronic use.

## Introduction

Respiratory diseases such as asthma and COPD are characterized by airway inflammation, airway obstruction and, in asthma, increased airway hyperresponsiveness (AHR) which is manifested by excessive constriction of airway smooth muscle (ASM) [1]. Short-acting β2-AR agonists (SABAs), such as salbutamol, are currently the most effective bronchodilators and are widely used as rescue medication. Long-acting β2-AR agonists (LABAs), such as salmeterol and formoterol, taken twice daily or other once-daily bronchodilators, such as vilanterol, indacaterol or olodaterol, are used on a chronic basis in combination with anti-inflammatory agents [2]. β2-AR agonists mediate relaxation of airway smooth muscle through signaling of Gs protein coupled β2-adrenoceptors (GPCR) which activates the enzyme adenylyl cyclase (AC) to produce cyclic adenosine 3’,5’-monophosphate (cAMP), which in turn activates protein kinase A (PKA). Phosphorylation of myosin light chain kinase and other specific target proteins by PKA induces smooth muscle relaxation by reducing intracellular [Ca^2+^] concentration and decreasing Ca^2+^-sensitivity of the contractile elements [3]. However, human studies have shown that excessive use of β2-AR agonists can induce desensitization of β2-adrenoreceptors [4], which increases tolerance and subsequently reduces responsiveness to β2-AR agonist stimulation [5]. The need to find compounds that provide therapeutic relief but act through alternative intrasignaling pathways from different classes of receptors could improve the treatment of obstructive airway diseases. Several new classes of bronchodilators including PDE4 subtype specific inhibitors, bitter tastants and chloride channel modulators are being considered [6]. In the present study we are proposing a novel potential therapeutic target.

ASM-024 is a readily absorbed synthetic homopiperazinium compound which does not cross the blood brain barrier with activity at the nicotinic and muscarinic receptors levels. In preclinical studies, ASM-024 attenuated airway resistance in mice and promoted relaxation of methacholine and histamine-induced contraction of mouse and guinea pig tracheas as well as dog and human bronchi [7].

Acetylcholine receptors are expressed on numerous cell types, both neuronal and non-neuronal [8]. In addition to muscarinic receptors, the expression of several nicotinic receptor subtypes on mouse tracheal [9] and rat airway smooth muscle cells [10] was demonstrated. Experiments using whole cell voltage clamp experiments revealed that ASM-024 inhibits acetylcholine- and nicotine- evoked responses on human α3β4 and α7 subtypes expressed in Xenopus oocytes, indicating a potential antagonist effect on certain nicotinic receptor subtypes [11]. However, when co-applied with the type II α7 positive allosteric modulator, PNU-120596, ASM-024 appears to function as an agonist and effectively activates the α7 ion channel. Compounds with similar properties have been defined as “silent agonists” and were reported to present anti-inflammatory effects at the α7 nAChR level that is mediated by a signal transduction pathway independent of ion current [12]. In addition ASM-024 has been shown to have antagonist effects on acetylcholine-induced activation of the M1, M2, and M3 muscarinic receptors expressed in Xenopus oocytes (unpublished data). Moreover, activation of nAChRs and mAChRs has been associated with an increase of intracellular calcium concentration and regulation of several cellular functions through Ca^2+-^dependent mechanisms [13]. In this study, potential and differential modulatory role of ASM-024 in smooth muscle cell calcium regulation was investigated. The objective of this study was to verify if ASM-024 could provide an additive smooth muscle relaxant effect to beta agonists, particularly under conditions of β2 adrenoreceptor desensitization.

## Materials and Methods

ASM-024 was provided by Asmacure Ltée. ASM-024, methacholine (Methapharm Inc.) and salbutamol (Ventolin, GlaxoSmithKline) were solubilized in appropriate aqueous solution. Formoterol (LKT Laboratories Inc.) and salmeterol (Tocris) were diluted in DMSO. Tissues and cells were exposed to 0.1 to 0.5% DMSO. Isometric tensions were acquired with the data acquisition System MP150 (Biopac Systems) via an isometric force transducer (Harvard apparatus). The data were digitalized and analyzed with AcqKnowledge 3.7.3 software.

This study was carried out in strict accordance with the recommendations in the Guide for the Care and Use of Laboratory Animals of the Canadian Council on Animal Care (CCAC) regarding acclimation, environmental enrichment, and analgesic and anesthesia procedures. The protocols were approved by the Committee on the Ethics of Animal Experiments of Université Laval. In two experiments, tissues from human donors were obtained after written informed consent in accordance with an Internal Review Board-approved protocol at IUCPQ Research Center (Institut universitaire de cardiologie et de pneumologie de Québec) in Québec, QC (Canada).

### Isometric assays

Isometric studies were performed on canine and human bronchi or guinea pig tracheas. Dog bronchi were isolated from the lung of euthanized healthy Mongrel dogs and tracheas were obtained from Hartley guinea pig. In two separate experiments. Human bronchi were isolated from the healthy lung tissue obtained from two patients undergoing resection for lung cancer. These tissues were obtained from the IUCPQ site of the Respiratory Health Network Tissue Bank of the FRQS (www.tissuebank.ca). The subjects were non-smokers or ex-smokers for more than 6 months with normal respiratory functions.

Tracheal or bronchial preparations, devoid of adherent connective tissue and cut in 0.3–0.5 cm long segments, were suspended in 5 mL organ baths containing Krebs solution. Krebs composition in mM was: NaCl 118, KCl 4.7, KH_2_PO_4_ 1.18, MgSO_4_ 1.18, NaHCO_3_ 25, glucose 5.6, CaCl_2_ 2.5. The tissues were maintained at 37°C and continually gassed with 5% CO_2_ in O_2_. A passive tension of 0.5 g was applied to guinea pig tracheas and dog bronchi and 1 g was applied for human bronchial preparations. Before the beginning of each experiment, tension was maintained for a 30–60 minutes equilibration and washing period until it achieved a steady state.

### Additive effects of ASM-024 on beta2-agonists airway smooth muscle relaxation in dog and human bronchi

Salbutamol-induced relaxation was performed with dog bronchial preparations contracted with methacholine (10^-5^ M); cumulative doses of salbutamol (10^-7^ to 10^-4^ M) were added and changes of tension recorded. When no further relaxation was obtained, a single maximal dose of 10^-3^ M ASM-024 was added to the preparations. Two similar experiments were conducted with human bronchi.

At the end of the assay, the tissues that were bronchodilated with ASM-024 were vigorously washed and exposed again to methacholine, to demonstrate that of ASM-024 had no effect on tissue viability.

### β2-adrenoreceptor desensitization of guinea pig tracheas

Contraction of tracheal guinea pig preparations was induced with methacholine (10^-5^ M). When contraction had reach a plateau, tracheal preparations were exposed to sub-maximal concentrations of either salbutamol (5 x 10^-8^ M), salmeterol (2 x 10^-6^ M), formoterol (10^-9^ M) or to the appropriate control vehicle for 60 minutes, in order to define the full relaxation induced by each agonist. Tracheal preparations were then washed intensively to remove methacholine and immediately re-exposed to the same concentration of β2-AR agonist or control vehicle for an additional 3 hours (salbutamol and salmeterol) or 18 hours (formoterol). After additional extensive washings, tracheal preparations were contracted again with 10^-5^ M methacholine and β2-AR desensitization was assessed by exposure of each preparation to salbutamol (5 x 10^-8^ M).

#### Relaxant Effects of ASM-024, on beta2-Agonist Desensitized Guinea-Pig Tracheas.

β2-AR-desensitized tracheal tissues or vehicle control treated tissues were contracted with methacholine (10^-5^ M), desensitization was tested by exposure to salbutamol for 10 minutes, then the tissues were exposed to cumulative concentrations of ASM-024 (10^-7^ M to 10^-3^ M).

#### Effect of Pre Exposure of Guinea-Pig Tracheas to ASM-024

In order to determine the effect of long-term exposure of tracheal preparations to ASM-024 and potential loss of efficacy upon subsequent application, some tissues were exposed to a sub-maximal concentration of ASM-024 (5x10^-5^ M) for a total of 18 hours as described for β2-AR agonists. After extensive washings, tracheal preparations were re-contracted with methacholine 10^-5^ M and exposed to cumulative concentrations of ASM-024 (10^-7^ M to 10^-3^ M).

### Evaluation of ASM-024 effect on adenylate cyclase pathways

In some assays, methacholine pre-contracted guinea-pig tracheas were treated for 15 minutes with 10^-4^ M SQ22,536, an adenylate cyclase inhibitor, before the addition of cumulative concentrations of ASM-024 (10^-6^ M to 10^-3^ M).

#### cAMP measurements

Human bronchial smooth muscle cells (hBSMC) were obtained from Lonza Inc. and were maintained in DMEM supplemented with 10% fetal bovine serum, non-essential amino acid and penicillin/streptomycin. Cells were used between passages 4–10 for all experiments. cAMP production was assessed by stimulation of 5x10^5^ cells with log increasing concentrations of ASM-024 (10^-7^ M to 10^-3^ M), salbutamol (10^-4^ M), formoterol (10^-8^ M) or forskolin (10^-5^ M). Measurements of intracellular cAMP concentrations were performed using the cAMP assay kit (R and D Systems) according to the manufacturer instructions. Results are express as % of cAMP produced by cells exposed to forskolin, an adenylyl cyclase activator.

#### Measurement of transient calcium mobilization from intracellular stores

Intracellular Ca^2+^ ([Ca^2+^]i) levels were determined with the FluoForte calcium assay kit (Enzo Life Sciences). Normal HBSMC (Lonza) were cultured in 96 wells plate and used at 90–95% confluence. Cells were pretreated with increasing concentrations of ASM-024 (10^-5^ to 10^-3^ M) for 10 minutes. Time-response curves of [Ca^2+^]i signal were recorded in real-time monitoring of fluorescence intensity (excitation: 490 nm, emission: 525 nm) before and after challenge with 10^-5^ M methacholine or histamine. The resulting signal was quantified by subtracting the basal fluorescence obtained before challenge from the maximum peak heightafter challenge. Results are expressed as the percentage of control cells fluorescence.

### Statistical analyses

All data are presented as means ± standard error of the mean, and statistical significance were determined by ANOVA followed by post-hoc tests. Data were considered statistically significant when *p* < 0.05. Isometric assays results are expressed as % of maximal methacholine-induced contraction. EC_50_ values were estimated by linear regression of individual concentration-responses curves using XLfit (IDBS).

## Results

### ASM-024 increased *in vitro* relaxation response to maximally effective concentration of short—acting beta-2 agonists in dog and human bronchi

**Fig 1:**
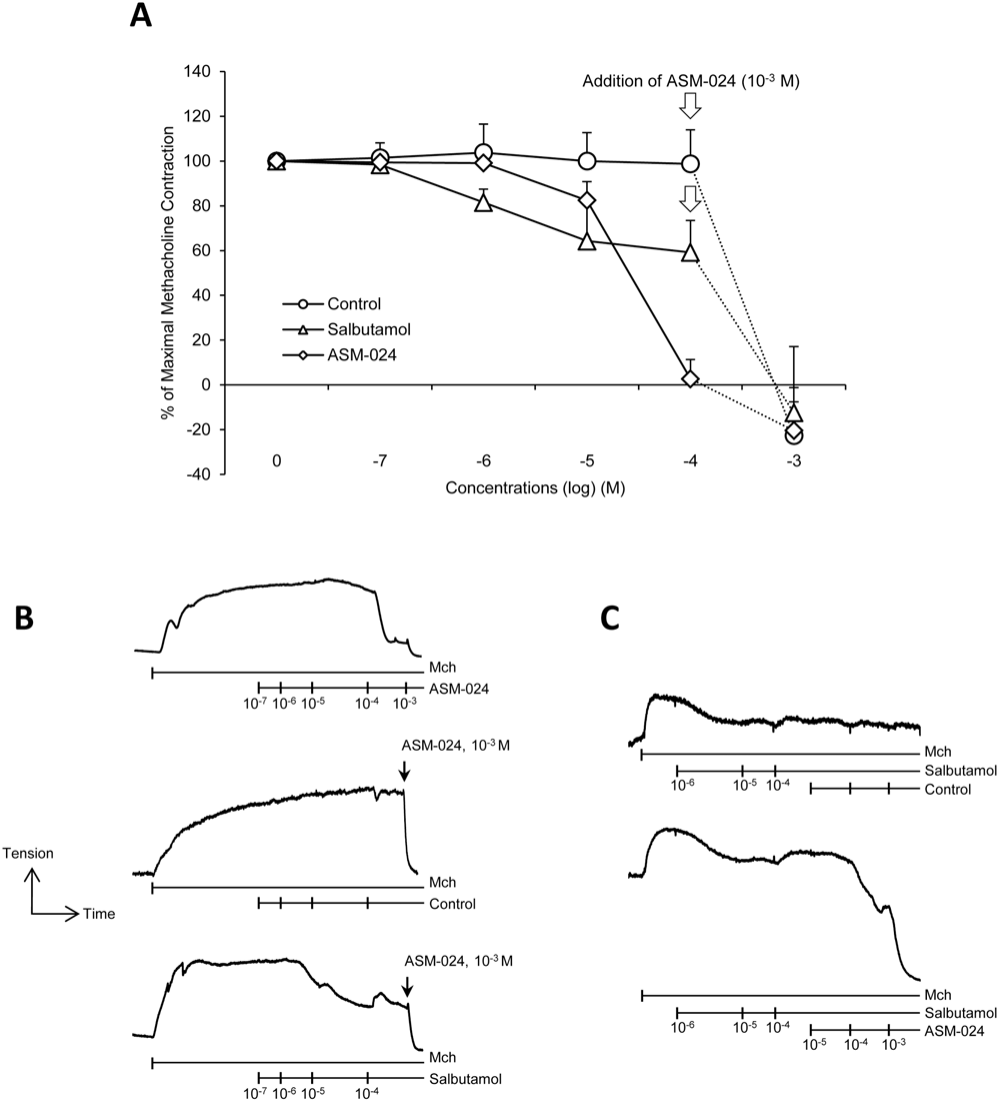
Additive effects of ASM-024 on beta2-agonists airway smooth muscle relaxation in dog bronchi. A) Dog bronchial preparations were contracted with 10–5 M methacholine and cumulative concentrations of ASM-024 or salbutamol added. ASM-024-induced bronchial relaxation required higher concentrations than the β2-AR agonist salbutamol (EC50 = 45.21 ± 13.74 μM for ASM-024 vs EC50 = 1.75 ± 1.83 μM for salbutamol). Salbutamol decreased the tension of precontracted dog bronchi by a maximum of 53%; the addition of 10–3 M ASM-024 further relaxed bronchial rings to below the applied basal tension. B) Representative tension recording depicting relaxation of dog bronchi following cumulative concentrations of salbutamol and ASM-024. n = 4–6 bronchial preparations from 3 different dogs. C) Representative tension recording showing that salbutamol, at 10–6 M maximally decreased the tension of contracted human bronchi; the addition of 10–4 M and 10–3 M ASM-024 to the maximal effective concentration of salbutamol increased relaxation to below the applied basal tension.

In isometric *in vitro* studies, ASM-024 caused a dose-related relaxation of dog bronchi contracted with methacholine with a rapid and complete relaxation observed at 10^-4^ M and 10^-3^ M. Salbutamol decreased the tension of precontracted dog bronchi at lower concentrations but a plateau was reached at about 50% relaxation; the subsequent addition of 10^-3^ M ASM-024 to the maximal effective concentration of salbutamol further increased the relaxation and elicited the same extent of relaxation as ASM-024 (Fig. 1).

Salbutamol at 10^-6^ M decreased the tension of contracted human bronchi preparations; no further dilation was obtained after cumulative concentrations of 10^-5^M and 10^-4^M. The addition of 10^-4^M and 10^-3^M ASM-024 to the maximally effective concentration of salbutamol increased relaxation to below the applied basal tension (Fig. 1). At the end of each assay, the bronchi segments are washed vigorously and re-contracted with methacholine to demonstrate the viability of the tissues.

### ASM-024 induces smooth muscle relaxation of β2-AR desensitized tracheas

**Fig 2:**
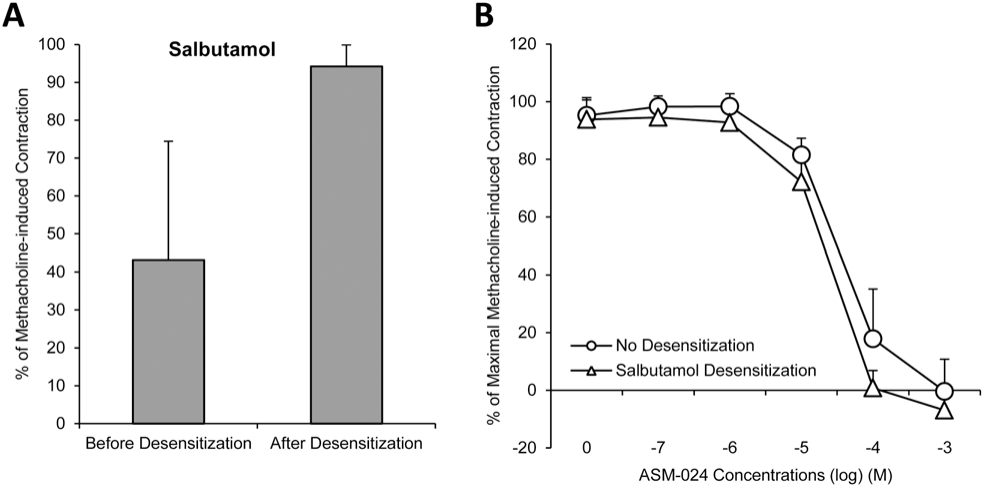
Relaxant Effects of ASM-024, on Salbutamol Desensitized Guinea-Pig Tracheas. A) Intact guinea pig tracheas were studied before or after 4 h of exposure to the β2 agonist salbutamol. A reduced relaxation response to the β2 agonist was observed after 4 hour exposure to salbutamol (p <0.05). B) No significant difference was observed on the relaxation response curves of ASM-024 on intact or salbutamol desensitized tracheas. EC50 = 40 ± 9 μM for intact tracheas; EC_50_ = 25 ± 11 μM for desensitized tracheas; values are means ± SD, n = 4.

**Fig 3:**
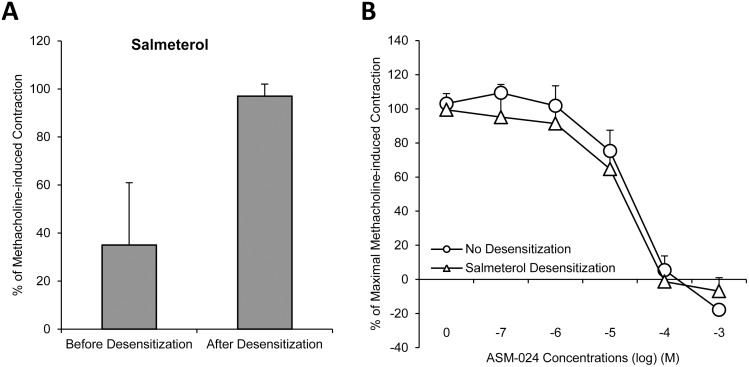
Relaxant Effects of ASM-024, on Salmeterol Desensitized Guinea-Pig Tracheas. A) Intact guinea pig tracheas were studied before and after 4 h of exposure to the long acting β2 agonist salmeterol. A reduced relaxation response to salbutamol is observed after 4 hour exposure to salmeterol (p <0.05). B) No significant difference was observed on the relaxation response curves of ASM-024 on intact or salmeterol desensitized tracheas. n = 4.

**Fig 4:**
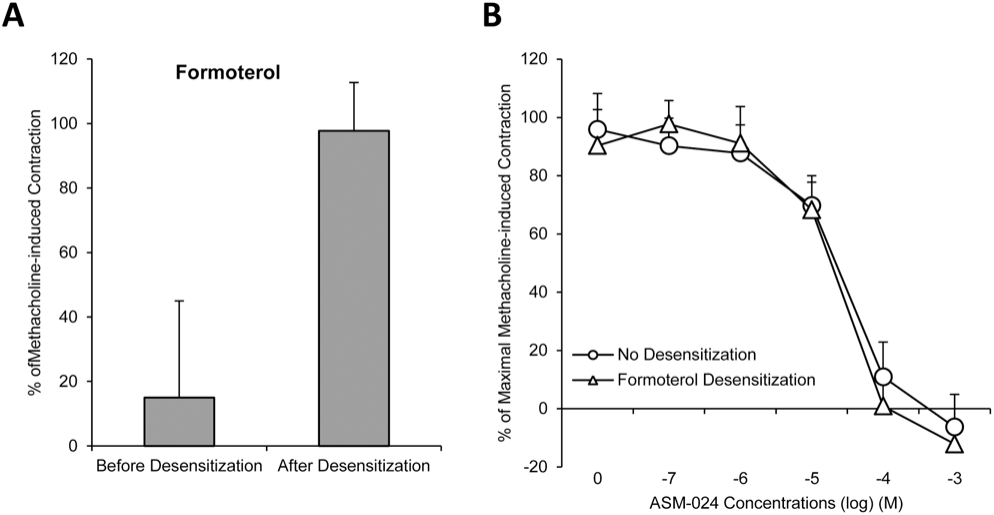
Relaxant Effects of ASM-024, on Salmeterol Desensitized Guinea-Pig Tracheas. A) Intact guinea pig tracheas were studied before and after 18 h of exposure to the long acting β2 agonist formoterol. A reduced relaxation response to salbutamol is observed after 18 hour exposure to formoterol (p <0.05). B) No significant difference was observed on the relaxation response curves of ASM-024 on intact or salmeterol desensitized tracheas. n = 4.

Guinea pig trachea unresponsiveness to salbutamol is demonstrated following a 4-hour incubation with either salbutamol (Fig. 2(A)) or salmeterol (Fig. 3(A)) while an 18-hour incubation was required to achieve the same reduced responsiveness with formoterol (Fig. 4(A)).

Cumulative concentrations of ASM-024 (10^-7^ M to 10^-3^ M) were tested on intact or beta2-adrenoreceptor tracheal preparations desensitized by salbutamol, salmeterol or formoterol. Relaxation response to ASM-024 on intact or adrenoreceptor desensitized tracheas was similar in each case (Fig. 2(B), Fig. 3 (B) and Fig. 4 (B)).

**Fig 5:**
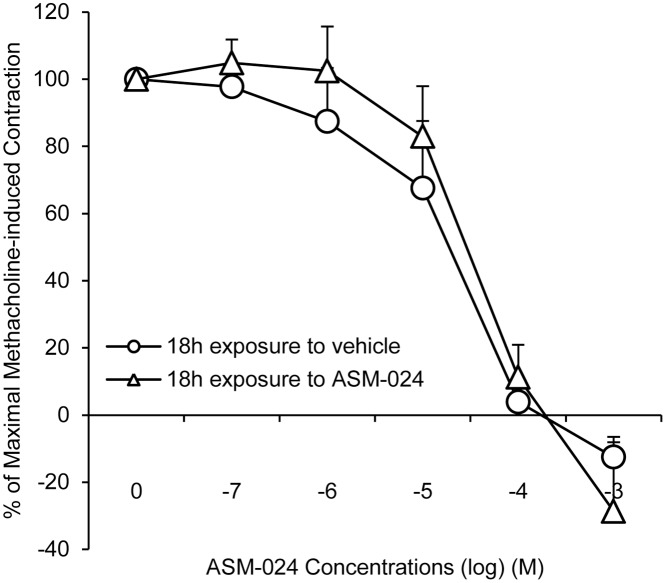
Effect of ASM-024 Pre-Exposure on the Relaxation Response to ASM-024. Guinea pig tracheas were pre incubated for 18h with 5x10-5 M of ASM-024, a concentration that induces 50% relaxation. Prolonged exposure of tracheas to ASM-024 had no significant effect on the responsiveness of the tracheas to subsequent treatment. n = 3–4.

Prolonged exposure of tracheas to ASM-024 did not induce significant hyporesponsiveness to subsequent treatment with the compound (Fig. 5). Compared to control vehicle-treated tracheas, ASM-024 inhibited the natural increase in tissue responsiveness to methacholine over time.

### Potential cAMP Involvement

**Fig 6:**
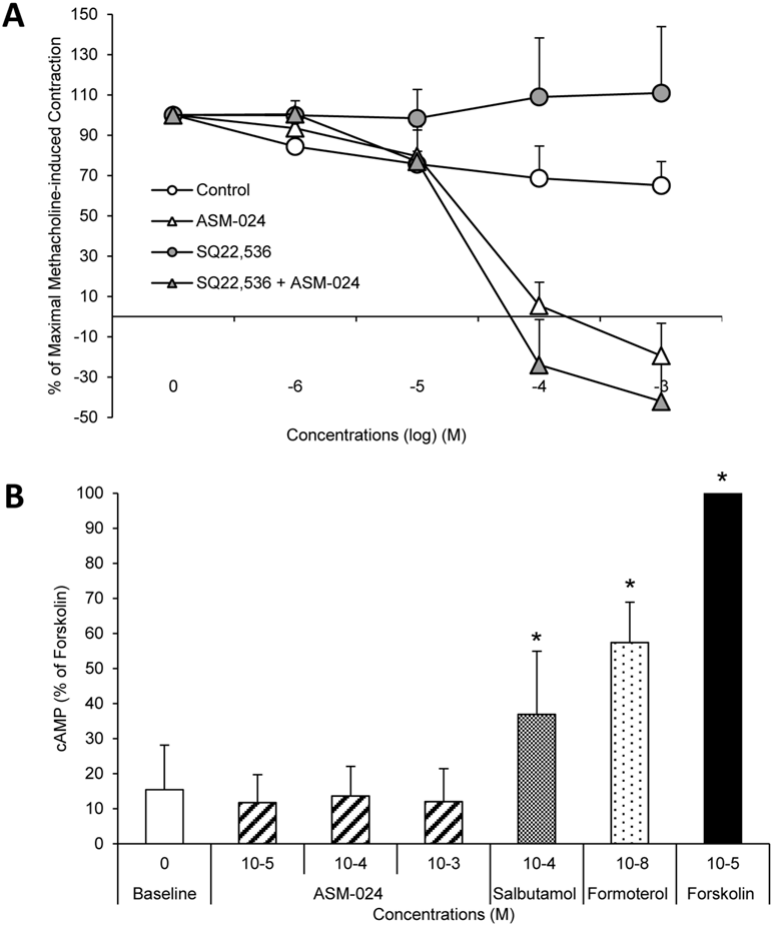
cAMP-Independent Mechanism in the Relaxant Effect of ASM-024. A) Blockade of cAMP synthesis with adenylate (SQ 22,536) cyclase inhibitors has no effect on ASM-024-induced relaxation. B) ASM-024 did not induce significant cAMP production in hBSMC cells. n = 4.

To investigate whether ASM-024 induces a downstream mechanism involving cAMP stimulation, tracheas were treated for 15 minutes with 10^-4^M SQ22,536, an adenylate cyclase inhibitor before the addition of cumulative doses of ASM-024. The blockade of cAMP synthesis, by SQ22,536 did not affect ASM-024-induced relaxation (Fig. 6(A)); EC50 = 36 ± 5 μM. Moreover, incubation of HBSMC cells with ASM-024 did not induce significant production of cAMP compared to salbutamol or formoterol treated cells (B).

### Effect of ASM-024 on transient Ca^2+^ mobilization from intracellular stores

**Fig 7:**
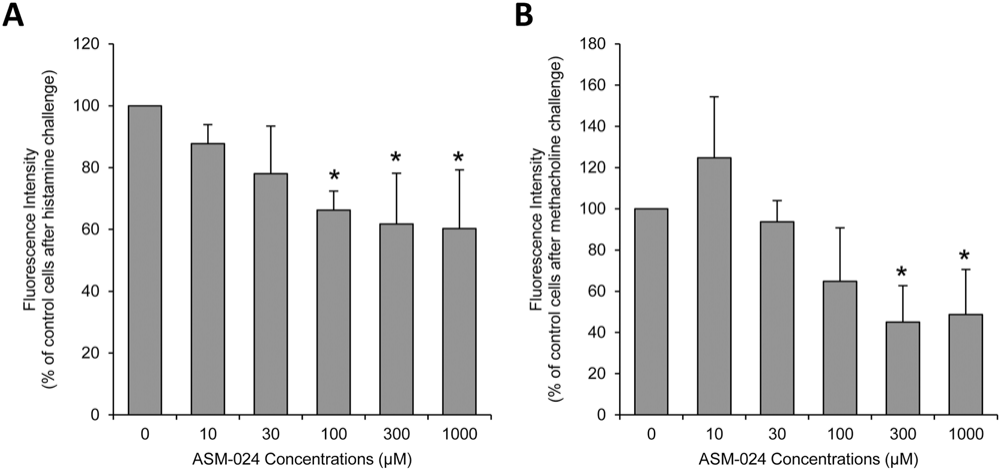
In Vitro Effect of ASM-024 on Intracellular Calcium Rise of Stimulated Human Bronchial Smooth Muscle Cells. Pre-incubation with ASM-024 for 10 minutes decreases [Ca2+]i rise in cells stimulated with 10–5 M histamine (A) or methacholine (B). EC_50_ = 73 ± 66μM for histamine and 107 ± 52 μM for methacholine, n = 4, *p<0.05.

Fifteen minutes incubation of hBSMC with ASM-024 alone had no effect on [Ca^2+^]i. However, a 10 minute pre-treatment with ASM-024 decreased [Ca^2+^]i rise in cells challenged with 10^-6^ M histamine or methacholine (Fig. 7).

## Discussion

Previous *in vitr*o isometric studies have shown that ASM-024 inhibited the contractile response to methacholine and histamine in a dose-dependent and reversible manner and had a protective effect on methacholine-induced constriction [7]. In this study, ASM-024 has demonstrated *in vitro* additive effects with salbutamol in isolated intact dog and human bronchi when added to maximally effective concentrations of salbutamol to even below the applied initial tension. In addition, ASM-024 was able to maintain its relaxation capacity on beta2-adrenoreceptor desensitized guinea pig tracheas, whereas prolonged exposure to ASM-024 did not affect its relaxant properties. Although the relaxation in response to ASM-024 required higher concentrations than β2 agonists, ASM-024 could elicit a complete dilation following the maximally effective concentration of SABA in dog and human bronchi. Dog and human bronchi were used to demonstrate the potential additive efficacy because, unlike guinea pig tracheas, they have only a partial response to β2 agonists. The additive effect on human bronchi demonstrates the relevance and potential application of this finding in humans. Since dog and human bronchi are not easily available, guinea tracheal preparations were used in the desensitization experiments and subsequent loss of responsiveness to β2 agonists. The concentrations used to achieve pharmacological responses *in vitro* were relatively high, however we have previously shown that doses used in *in vivo* mouse studies were lower and well within tolerated range [7]. This apparent discrepancy between *in vitro* and *in vivo* doses may be related to the rapid desensitization rate of the nicotinic receptor upon binding of the ligand, essentially resulting in the need for greater and continuous exposure to higher concentrations in order to achieve an *in vitro* pharmacological effect whereas recovery from the desensitized state may occur more rapidly *in vivo* [14].

β2 agonists and ASM-024act on two different classes of receptors: relaxation of smooth muscle following stimulation of beta-adrenergic receptors results from an increase in cAMP that stimulates a protein kinase which then phosphorylates a target proteins leading to relaxation by decreasing the intracellular concentration of Ca^2+^ [15]; ASM-024 relaxant effect appears to be cAMP-independent as demonstrated by the lack of cAMP accumulation in ASM-024 treated HBSMC. Moreover, blockade of cAMP synthesis by an adenylate cyclase inhibitor at a concentration known to inhibit guinea pig trachea relaxation [16] had no effect on ASM-024-induced relaxant properties using the same model.

As mentioned earlier, chronic activation of β2-adrenoreceptor is associated with loss of functional response and decreased clinical efficacy. In this study we have shown that a 4-hour *in vitro* pre-exposure of guinea pig tracheas with either salbutamol or salmeterol is sufficient to reduce responsiveness upon restimulation with the short-acting salbutamol. For formoterol, a pre-exposure of 18h was required to achieve a similar loss of responsiveness. Similar differences between various β2 agonists to induce β2-adrenoreceptor desensitization were reported and are probably due to differences in retention time or accumulation of agonists at the receptor site [17].

As indicated by standard radioligand receptor binding competition assays, ASM-024 is a quaternary ammonium compound with an affinity in the low micromolar range for the agonist binding site of the various nicotinic receptor subtypes. These early investigations (unpublished data) suggest that ASM-024 binds within the receptor pore and blocks the channel in a noncompetitive fashion. ASM-024 is a modulator of cholinergic receptor function and although its mechanism of action is not yet fully understood, it appears to functions as a non-competitive inhibitor of α7 and α3β4 nicotinic receptor ion channel function [11]. These findings suggest that ASM-024 mediates smooth muscle relaxation through a different target and signaling pathway than currently used beta2-adrenergic receptor agonists.

Chronic treatment of asthma and COPD often requires increasingly higher doses of inhaled corticosteroids and LABAs. Chronic use of certain LABAs can increase airway hyperresponsiveness and reduce response to SABAs when needed for acute use [18]. Also, maintenance treatment with LABAs (with or without ICS) has been shown to induce tolerance to their bronchoproptective effects and cross-tolerance to the reliever effects of the SABAs. Thus, there is a need for therapies with different mechanisms of action that could offer an adjunctive benefit to current treatment. ASM-024 or other components of this class could potentially be considered as adjunct agents to reduce the dose and the use of β2 agonists in patients and potentially enhance the control of the patient’s disease.
